# Prospective analysis of sleep characteristics, chronotype, and risk of breast cancer in the california teachers study

**DOI:** 10.1007/s10552-023-01817-5

**Published:** 2023-11-09

**Authors:** Julie Von Behren, Debbie Goldberg, Susan Hurley, Jessica Clague DeHart, Sophia S. Wang, Peggy Reynolds

**Affiliations:** 1 Department of Epidemiology and Biostatistics, University of California San Francisco, San Francisco, CA USA; 2 School of Community and Global Health, Claremont Graduate University, Claremont, CA USA; 3 Division of Health Analytics, Department of Computational and Quantitative Medicine, Beckman Research Institute, City of Hope Comprehensive Cancer Center, Duarte, CA USA

**Keywords:** Breast cancer risk, Sleep, Chronotype, Circadian disruption, Circadian rhythm

## Abstract

**Purpose:**

Poor sleep quality and evening chronotype were associated with increased risk of breast cancer in a previous retrospective study in the California Teachers Study (CTS). The present analysis examines these sleep factors prospectively in the same cohort of women.

**Methods:**

From the CTS, we included 1,085 incident breast cancer cases and 38,470 cancer-free participants from 2012 through 2019. We calculated time at risk and used Cox proportional hazards regression models to estimate the hazard ratios (HRs) and control for risk factors such as age, race, body mass index, family history of breast cancer, and reproductive history. The sleep factors examined were quality, latency, duration, disturbance, and sleep medication use, based on a shortened version of the Pittsburgh Sleep Quality Index, as well as chronotype (preference for morning or evening activity). This analysis was limited to women who were post-menopausal at the time they answered these sleep-related questions.

**Results:**

Measures of sleep quality did not appear to be associated with subsequent breast cancer risk. The HR for evening chronotypes compared to morning chronotypes was somewhat elevated (HR 1.19, 95% CI 1.04, 1.36).

**Conclusion:**

While the measures of sleep quality and duration were not associated with post-menopausal breast cancer risk in this prospective analysis, the modestly elevated risk observed for evening chronotypes was consistent with the prior retrospective analysis.

**Supplementary Information:**

The online version contains supplementary material available at 10.1007/s10552-023-01817-5.

## Introduction

Shift work and night work cause circadian disruption and have been recognized as probable human carcinogens by the International Agency for Research on Cancer (IARC) [1]. Chronotype, which is an individual’s preference for morning or evening activity also impacts circadian rhythms and women with evening chronotypes (means prefer evening time) may be at increased risk for breast cancer [2–5]. Recent studies have also investigated various aspects of sleep quality, such as difficulty sleeping and frequent waking, but no consistent patterns with breast cancer risk have been observed [6–11]. Both long and short sleep durations have been examined in numerous breast cancer studies as well, with no clear pattern of association [7, 12–17]. Some of the heterogeneity in the risk estimates across the various studies to date may be due to the different study designs, which include case–control studies, as well as retrospective and prospective cohorts.

The underlying physiological mechanisms that may drive associations between sleep factors and breast cancer risks are not completely understood and are likely complex [18]. Many hypotheses about different pathways have been proposed, such as melatonin changes, cellular damage via oxidative stress, altered metabolic function, and inflammation [18]. These biological mechanisms could operate through both sleep disturbance and circadian disruptions.

Breast cancer is the most commonly diagnosed cancer in women in the United States, aside from skin cancers [19]. Most female breast cancers (71%) are diagnosed in women ages 55 and older, generally considered the post-menopausal time period [20]. We have studied sleep and chronotype in the California Teachers study (CTS), a cohort of women enrolled in 1995 and whose members are now predominantly over age 65 years. Increased post-menopausal breast cancer risk was modestly associated with evening chronotype in a recent retrospective analysis in these women [21]. Sleep quality, latency, and disturbance were also associated with increased breast cancer risk [22]. One of the limitations of the retrospective analysis was that the questions about sleep and chronotype were asked after the cancer diagnosis, which could lead to recall differences between the women with and without the disease. The present analysis examines sleep factors and chronotype prospectively in the same cohort of women to determine if the associations are still evident when using a prospective follow-up study design.

## Materials and methods

### Study population

The California Teachers Study (CTS) was established in 1995 when the initial questionnaires were sent to active and retired females enrolled in California’s State Teachers Retirement System. A total of 133,477 women completed the first questionnaire that included information on pregnancy history, personal and family medical history, health behaviors, body size, smoking, diet, and other lifestyle factors, as previously described [23]. Five subsequent questionnaires were administered to collect additional information on topics of emerging interest. Sleep duration, sleep quality, sleep medication use, and chronotype were assessed on the fifth CTS Questionnaire (Q5), administered in 2012–2013. There were 65,298 respondents to Q5, which was approximately 60% of the initial cohort that was still alive and eligible to participate. The use of human subjects in the CTS was reviewed and approved by the Institutional Review Boards at all participating institutions and by the Committee for the Protection of Human Subjects at the California Health and Human Services Agency. Informed consent was obtained from the participants.

### Inclusion criteria and identification of breast cancer cases

From the 65,298 respondents to the fifth CTS Questionnaire, we excluded the following participants: three women for administrative reasons, three women who died before the completion date of the questionnaire, 3,304 women who did not live in California at baseline, 6,852 women who did not live in California at the time of Q5, and 11,522 women with an invasive cancer of any type diagnosed prior to Q5. We also limited this analysis to post-menopausal women at the time of Q5, resulting in the exclusion of 4,059 additional participants. Cancer cases were identified through annual linkages to the California Cancer Registry files. We included invasive breast cancer cases that were diagnosed after the participant completed the fifth questionnaire (from 2012 through 2019). The cases included in this analysis did not overlap with the cases included in the previous retrospective study. The breast cancer cases were based on the SEER site recode 26000 [24]. The final study population included in this analysis was 39,555 women, with 1,085 cases and 38,470 cancer-free participants.

### Sleep quality questions

The sleep questions were based on a shortened version of the Pittsburgh Sleep Quality Index (PSQI) [25], which has been used in a variety of health outcome studies. We included five of the original 19 PSQI questions about usual sleep habits during the past month. These questions assessed overall sleep quality, latency (how long it takes to fall asleep), duration (hours per night), disturbance (trouble falling asleep, waking in the night, or waking too early), and sleep medication use. The full text of the CTS questionnaire is available online at https://www.calteachersstudy.org/_files/ugd/49684b_675b2770f02646cda6df88a3cb6e5187.pdf. The 19 questions in the original full-length version of the PSQI are usually summarized into a global sleep index (GSI). We created a modified version of the GSI by scoring the five questions included on the CTS questionnaire. We assigned scores from 0 to 3, with 0 representing the best sleep and 3 representing the worst sleep and then added the individual scored components to create a total score that ranged from zero to 15 [22]. We then categorized the total score into four groups with the lowest scores representing the best overall sleep quality (scores ≤ 4) and the highest scores representing the worst sleep quality (score ≥ 9).

### Definition of chronotypes

The single chronotype question was based on the Horne-Ostberg Morningness-Eveningness Questionnaire [26]. This question asked the following: “One hears about ‘morning’ and ‘evening’ types of people. Which do you consider yourself to be?” The response choices were “definitely a morning type”, “more a morning than an evening type”, “neither a morning or an evening type”, “more an evening than a morning type”, or “definitely an evening type”. For this analysis “definitely a morning type” and “more a morning type” were combined into one category and “definitely an evening type” and “more an evening type’ were combined into one category.

### Statistical analysis

We used Cox proportional hazards regression models to estimate the hazard ratios (HRs) and 95% confidence intervals (CIs) of the associations between sleep characteristics and breast cancer diagnosis. We calculated the time at risk for each participant from the date of questionnaire 5 until the date of breast cancer diagnosis, date of death, or end of follow-up time (31 December 2019). Multivariable models included variables chosen by backward selection to identify covariates of interest. Initial models included age, race/ethnicity, family history of breast cancer, body mass index (BMI), physical activity, marital status, age at menopause, use of hormone replacement therapy, use of pain medication or nonsteroidal anti-inflammatory drugs, comorbidities reported at Q5 (diabetes, depression, chronic obstructive pulmonary disease, Parkinson’s disease, chronic fatigue syndrome, lupus, irritable bowel syndrome, Crohn’s disease, and multiple sclerosis), household income, education level, smoking history, alcohol consumption, age at menarche, pregnancy history, and breast feeding history. These factors were chosen a priori based on our previous breast cancer research in this cohort. Comorbidities were examined as a combined index score (total number of conditions) and then again with each comorbid condition separately. The backward selection forced the inclusion of age and race/ethnicity and kept variables with Wald chi-square < 0.05. For all models the remaining covariates were BMI and breast cancer family history. The analysis for each individual sleep characteristic excluded 431 participants who did not provide responses for all sleep questions and those with unknown values for that sleep variable. The analysis for chronotype excluded 535 participants who did not answer the chronotype question. Statistical analyses were conducted in SAS version 9.4 (SAS Institute, Cary, North Carolina) in the CTS Researcher Platform [27].

## Results

The mean follow-up time for the participants included in this analysis was 6.5 years. The average age of the participant at the time of completing the fifth questionnaire was 68 years and 87% were non-Hispanic white. The characteristics of this cohort have been previously described in detail [21, 22]. A higher proportion of the women with breast cancer reported a family history of the disease compared to women without breast cancer (24% and 16%, respectively). For BMI at the time the sleep questions were asked, about 34% of cases were overweight (BMI = 25–29 kg/m^2^) and 20% were obese (BMI ≥ 30 kg/m^2^). Among the non-cases, the prevalence of overweight and obesity was slightly lower, with 29% overweight and 19% obese. In general, the cases and non-cases were quite similar in terms of age, smoking status, alcohol consumption, and pregnancy history (Supplemental Table 1).

The distribution of sleep characteristics and chronotype by breast cancer status are shown in Table 1. Self-reported sleep quality was similar for the women with and without breast cancer. About 30% in both groups reported very good sleep quality and about 15% reported fairly bad or very bad sleep quality. Approximately 17% of both cases and non-cases reported more than 30 min to fall asleep each night during the past month (long latency). Frequent sleep disturbances (3 or more times per week) were reported for 21% of cases and 20% of non-cases. Both groups also reported similar average sleep duration, with 67% of cases and 68% of non-cases reporting 7 to 8 h of sleep per night. Only 5% in both groups reported 9 or more hours of sleep per night on average. Frequent use of sleep medications (more than 2 times per week) was reported by 14% of the cases and 13% of non-cases. The Global Sleep Index (GSI) scores from the modified PSQI were similar for cases and non-cases with 26% of cases and 25% of cancer-free participants having the worst overall sleep quality (GSI score 9 or higher). The prevalence of evening chronotypes was higher among cases than among non-cases (32% and 27%, respectively), whereas morning types were more common among non-cases (55% in cases and 57% in non-cases).

**Table 1:** Risk of post-menopausal breast cancer incidence associated with sleep characteristics and chronotype: prospective analysis in the California Teachers Study, cases diagnosed 2012–2019

Sleep characteristic	Level	Cases (%) *n* = 1,085	Non-cases (%) *n* = 38,470	Multivariable^*^ adjusted HR and 95% CI
Sleep quality	Very good	325 (30)	11,624 (31)	1.00 (ref)
	Fairly good	587 (55)	20,661 (54)	1.03 (0.90, 1.19)
	Fairly bad or very bad	156 (14)	5,582 (15)	1.02 (0.84, 1.23)
Sleep latency	< 15 min	534 (50)	17,753 (47)	1.00 (ref)
	16–30 min	361 (34)	13,732 (36)	0.88 (0.76, 1.00)
	31–60 min	143 (13)	4,817 (13)	0.97 (0.81, 1.17)
	> 60 min	35 (3)	1,557 (4)	0.77 (0.55, 1.09)
Sleep disturbance	Not during past month	237 (22)	8,497 (22)	1.00 (ref)
	< 1 time/week	331 (31)	12,345 (32)	0.97 (0.82, 1.15)
	1–2 time/week	272 (25)	9,411 (25)	1.04 (0.88, 1.25)
	≥ 3 times/week	229 (21)	7,646 (20)	1.08 (0.90, 1.30)
Sleep duration	< 5 h	30 (3)	993 (3)	1.08 (0.74, 1.59)
	5–6 h	264 (25)	9,296 (24)	1.02 (0.86, 1.21)
	7 h	439 (41)	15,812 (42)	0.98 (0.84, 1.14)
	8 h	280 (26)	9,807 (26)	1.00 (ref)
	≥ 9 h	53 (5)	1,804 (5)	0.99 (0.73, 1.33)
Sleep medication	Not during past month	752 (70)	26,242 (69)	1.00 (ref)
	< 1 time/week	107 (10)	4,120 (11)	0.91 (0.74, 1.11)
	1–2 time/week	58 (5)	2,239 (6)	0.91 (0.69, 1.18)
	≥ 3 times/week	154 (14)	5,093 (13)	1.00 (0.84, 1.19)
Global Sleep Index (GSI)	Lowest scores GSI 1–4 (better sleep)	317 (29)	10,892 (29)	1.00 (ref)
	GSI 5–6	249 (23)	9,282 (24)	0.93 (0.79, 1.10)
	GSI 7–8	198 (18)	7,356 (19)	0.94 (0.79, 1.13)
	Highest scores GSI 9–17 (worse sleep)	281 (26)	9,444 (25)	1.01 (0.86, 1.19)
Chronotype	Morning type/more morning than evening type	588 (55)	21,823 (57)	1.00 (ref)
	Neither morning/evening type	131 (12)	4,986 (13)	0.94 (0.78, 1.14)
	Evening type/more evening than morning type	343 (32)	10,230 (27)	1.19 (1.04, 1.36)

Analysis for each individual sleep characteristic excluded participants who did not provide responses for all sleep questions

Analysis for the chronotype variable excluded those who did not provide a response for the chronotype question

^*^ Risk estimates adjusted for age, race/ethnicity, BMI, and family history of breast cancer

Hazard ratios for the risk of post-menopausal breast cancer incidence associated with the sleep quality characteristics and chronotype are shown in Table 1. These risk estimates were adjusted for age, race/ethnicity, BMI, and family history of breast cancer. When we compared women who reported poor sleep quality to those who reported very good sleep quality, the HR was 1.02 (95% CI 0.84, 1.23). The HR for the longest sleep latency (> 60 min to fall asleep at night) was 0.77 (95% CI 0.55, 1.09) compared to women who reported falling asleep quickly (< 15 min). There was no difference in risk between the participants with the worst overall sleep scores (GSI ≥ 9) compared to the group with the best sleep scores (GSI ≤ 4), with a HR of 1.01 (95% CI 0.86, 1.19). The HRs for all of the categories within sleep disturbance, sleep duration, and sleep medication usage were also around one (ranging from 0.91 to 1.08) and none were statistically significant. The HR for the evening chronotypes compared to morning chronotypes was modestly elevated with a HR of 1.19 (95% CI 1.04, 1.36).

Given that the average age of this cohort is 68 years and the recommended nighty sleep is 7–8 h for people 65 years and older, we also examined average hours of nightly sleep by combining 7–8 h of sleep as the refence group, to align with the national sleep guidelines [28]**.** The results were unchanged. The HR for sleep durations of 9 or more hours was 1.00 (95%CI 0.75–1.33). The HR for 5–6 h was 1.04 (95% CI 0.90, 1.20) and HR for < 5 h of sleep was 1.10 (95% CI 0.76, 1.59).

Cases that occurred close to the time of filling out the questionnaire may not accurately reflect relationships between sleep patterns and cancer due to latency of the disease. Therefore, we conducted a sensitivity analysis excluding 182 cases that were diagnosed within one year of completing the relevant questionnaire (Q5). The results were generally the same for sleep quality, latency, duration, and medication, with confidence intervals that included one (data not shown). However, the HR for chronotype increased somewhat from 1.19 (95% CI 1.04, 1.36) to 1.32 (95% CI 1.14, 1.52).

## Discussion

The elevated risk of post-menopausal breast cancer among the women who were evening chronotypes compared to morning types in this prospective analysis was of the same magnitude as in our retrospective analysis in this cohort (HR 1.19 and Odds Ratio (OR) 1.20, respectively) [21]. In a retrospective analysis done in the Nurses’ Health Study II, another large cohort of U.S. women, evening chronotypes were at similarly increased risk for breast cancer (OR 1.15, 95% CI 0.98, 1.56), but the estimate was not statistically significant [3]. In addition, the nurse participants with no preference for morning or evening were also at increased risk (OR1.27, 95% CI 1.04, 1.56) [3]. Similarly, a case–control study of Danish military workers found increased breast cancer risks for women with nighttime preferences (OR 1.8, 95% CI 1.2, 2.9) as well as those with no preference (OR 1.6, 95% CI 1.0, 2.7) [2]. A modest protective effect was reported for morning chronotype in a large prospective breast cancer investigation in the United Kingdom (HR 0.95, 95% CI 0.93, 0.98) [5]. In a breast cancer case–control study from India, both morning and evening types had significantly elevated odds ratios compared to women with no preference [4].

In this current prospective analysis of the measures of sleep deficiency and quality in the CTS, no significantly increased breast cancer risks were associated with reportedly poorer sleep. This finding differs from the results of our previous retrospective analysis of breast cancer risk in the cohort where we found that the individual sleep components of quality, latency, and disturbance were all associated with increased breast cancer risks [22]. The summary Global Sleep Index based on the shortened PSQI was also previously associated with increased risk (HR 1.24, 95% CI 1.12, 1.38) for the highest scores compared to lowest scores [22]. There are several possible reasons for our different findings regarding the risks associated with sleep quality and the GSI. The previous retrospective analysis we conducted may have been subject to bias due to breast cancer possibly impacting self-reported sleep quality after diagnosis. In the present prospective analysis, the sleep questions were asked prior to breast cancer diagnosis which should be less biased, although some researchers have noted that breast cancer may affect sleep before it is clinically diagnosed (reverse causality) [7, 12]. Additionally, the sleep quality information used in both the prospective and retrospective analyses was self-reported at a single point in time and sleep habits change over time and with aging. The Global Sleep Index summarized five questions on the PSQI and weighted all dimensions of sleep equally making it difficult to interpret. The individual sleep components such as quality, disturbance, latency, and duration, may be more important to consider and more generalizable than using a composite index score.

In an analysis of the Sisters Study, another prospective cohort, White et al. found that women who reported having difficulty sleeping four or more nights per week had increased breast cancer risk (HR 1.32, 95% CI 1.09, 1.61) [6]. However, several other studies, including most of the previous studies that have examined various measures of sleep quality and breast cancer risks, have found no association [7–11]. These studies have included both cohort and case–control studies, and a variety of measures for self-reported sleep quality, including daytime napping, difficulty falling asleep, waking at night, sleep disturbances, and overall sleep quality.

Most of the published meta-analyses of the literature on sleep duration and breast cancer have found no risks associated with either short or long duration of sleep [7, 12–17]. In the current analysis, we also did not observe any increased risks associated with longer sleep duration. Previously, we assessed the prospective risk of breast cancer associated with sleep duration that was reported at the time of entry into the California Teachers Study (rather than as reported later on Q5 as in the present analyses). The HR for 10 or more hours of sleep per night compared to 7–9 h per night was elevated, but not statistically significant (1.25, 95% CI 0.93, 1.68) [29]. The inconsistent findings reported in the epidemiological literature on sleep and cancer could be due to the lack of standardized ways to assess sleep quality and sleep changes over the life-course [30]. Self-reported sleep duration has shown poor agreement with both actigraphy measures [31] and polysomnography (PSG) [32]. The consistency of association with evening chronotype in the California Teachers Study cohort, in contrast to that for sleep duration and quality measures, may be because chronotype is more stable over time than sleep duration and quality and therefore more accurately self-reported. In a recent commentary, Erren and Lewis wrote that epidemiological studies of sleep need to employ detailed questionnaires that assess details of sleep factors over time and these studies could benefit from the use of actimetry devices [30]. They also point out the growing need for ways to standardize all of these measures for comparisons across studies.

In addition to sleep quality and duration, having a chronic sleep disorder may be related to cancer risk. Both sleep apnea and insomnia have been associated with increased breast cancer risks in recent literature reviews [33, 34]. In a cohort study from Taiwan, the researchers looked at the presence of any type of clinically diagnosed sleep disorder and breast cancer risk and reported a Hazard Ratio of 1.17 (95% CI 0.98,1.39) [35]. Although we lacked information on sleep disorders for the present analysis, additional detailed sleep questions were asked on the sixth questionnaire (Q6) for the California Teachers Cohort (subsequent to the current analysis of Q5), which will allow for future assessments to utilize an insomnia index and other sleep disorders. Sleep patterns and circadian rhythms are influenced by many behavioral, biological, psychosocial, and environmental factors [18]. These relationships are difficult to tease out and will require both enhanced epidemiological methods and additional experimental investigations into the potential biological pathways [18]. Although direct mechanisms linking chronotype and circadian disruptions to breast cancer are not clearly established, circadian rhythms are involved in the regulation of many important biological processes such as DNA repair, immune function, and tumor suppression [36]. Many clock genes are involved in regulating circadian rhythms and they may affect cancer risks, however findings from genetic studies vary [37]. An interesting laboratory study subjected mice genetically prone to breast cancer to alternating light and dark cycles to cause chronic circadian rhythm disturbances and found the mice exhibited decreased suppression of breast tumors [38].

The primary strength of this analysis is that it was conducted prospectively in a well-characterized cohort of women with a comprehensive state-wide cancer registry for obtaining the case information. Additionally, there is extensive information available on individual breast cancer risk factors and the cohort database is routinely updated through additional linkages to hospital discharges, death records, and change-of-address information. However, this analysis was limited by the relatively short follow-up time (6.5 years) for 1,085 incident cases.

## Conclusion

In this prospective cohort analysis, we observed a modestly elevated post-menopausal breast cancer risk associated with evening chronotype. This finding was consistent with the prior retrospective analysis of chronotype in this same cohort of women. In contrast to the previous study however, measures of sleep quality and duration were not associated with breast cancer risk. These findings are generally consistent with the previous literature on these topics, where sleep duration and quality have had no clear patterns of association with breast cancer risks. Self-reported chronotype, which may be more stable in an individual over time than sleep quality, has been shown to be more consistently related to elevated breast cancer risks and is an important factor to consider in epidemiological studies that assess sleep patterns and circadian disruptions.

### Supplementary Information

Below is the link to the electronic supplementary material.

Supplementary file1 (DOCX 54 KB)

## Data Availability

All of the data associated with this publication and in the California Teachers Study are available for research use. Information is available at https://calteachersstudy.my.site.com/researchers/s/
