# Supplementary material for: Prospective analysis of sleep characteristics, chronotype, and risk of breast cancer in the california teachers study
Source: Cancer Causes Control. 2023 Nov 9;35(4):597–604. doi: 10.1007/s10552-023-01817-5 (PMC10960753; doi:10.1007/s10552-023-01817-5)
Supplement: Supplementary file 1 — Supplementary file1 (DOCX 54 KB) [file 10552_2023_1817_MOESM1_ESM.docx]

**Supplemental Table 1. Covariate characteristics by breast cancer diagnosis in the California Teachers Study, cases diagnosed 2012-2019.**

| **Covariate** | **cases** | | **non-cases** | |  | **Total** | |
| --- | --- | --- | --- | --- | --- | --- | --- |
|  | **N** | **%** | **N** | **%** |  | **N** | **%** |
|  |  |  |  |  |  |  |  |
| All | 1,085 | 100 | 38,470 | 100 |  | 39,555 | 100 |
|  |  |  |  |  |  |  |  |
| **Covariates included in fully adjusted model:** |  |  |  |  |  |  |  |
|  |  |  |  |  |  |  |  |
| Age (years) |  |  |  |  |  |  |  |
| 39-49 | 14 | 1 | 726 | 2 |  | 740 | 2 |
| 50-59 | 152 | 14 | 6,739 | 18 |  | 6,891 | 17 |
| 60-69 | 442 | 41 | 15,763 | 41 |  | 16,205 | 41 |
| 70-79 | 328 | 30 | 9,646 | 25 |  | 9,974 | 25 |
| 80-89 | 131 | 12 | 4,813 | 13 |  | 4,944 | 12 |
| 90-106 | 18 | 2 | 783 | 2 |  | 801 | 2 |
|  |  |  |  |  |  |  |  |
| Race |  |  |  |  |  |  |  |
| Non-White | 114 | 11 | 4,976 | 13 |  | 5,090 | 13 |
| White | 971 | 89 | 33,494 | 87 |  | 34,465 | 87 |
|  |  |  |  |  |  |  |  |
| Body Mass Index (kg/m^2^) |  |  |  |  |  |  |  |
| 15-24 | 448 | 41 | 18,283 | 48 |  | 18,731 | 47 |
| 25-29 | 369 | 34 | 11,067 | 29 |  | 11,436 | 29 |
| 30-54.8 | 212 | 20 | 7,119 | 19 |  | 7,331 | 19 |
| Unknown | 56 | 5 | 2,001 | 5 |  | 2,057 | 5 |
|  |  |  |  |  |  |  |  |
| Family history of breast cancer |  |  |  |  |  |  |  |
| No | 803 | 74 | 31,109 | 81 |  | 31,912 | 81 |
| Yes | 255 | 24 | 6,288 | 16 |  | 6,543 | 17 |
| Unknown | 27 | 2 | 1,073 | 3 |  | 1,100 | 3 |
|  |  |  |  |  |  |  |  |
| **Covariates considered but not included in final model:** |  |  |  |  |  |  |  |
|  |  |  |  |  |  |  |  |
| Smoking history |  |  |  |  |  |  |  |
| Never | 706 | 65 | 25,852 | 67 |  | 26,558 | 67 |
| Former | 336 | 31 | 11,008 | 29 |  | 11,344 | 29 |
| Current | 38 | 4 | 1,447 | 4 |  | 1,485 | 4 |
| Unknown | 5 | <1 | 163 | <1 |  | 168 | <1 |
|  |  |  |  |  |  |  |  |
| Smoking pack-years |  |  |  |  |  |  |  |
| Never smokers | 706 | 65 | 25,852 | 67 |  | 26,558 | 67 |
| ≤10 | 192 | 18 | 6,701 | 17 |  | 6,893 | 17 |
| 11-20 | 66 | 6 | 2,242 | 6 |  | 2,308 | 6 |
| 21-30 | 39 | 4 | 1,186 | 3 |  | 1,225 | 3 |
| ≥31 | 45 | 4 | 1,262 | 3 |  | 1,307 | 3 |
| Unknown | 37 | 3 | 1,227 | 3 |  | 1,264 | 3 |
|  |  |  |  |  |  |  |  |
| Alcohol consumption (grams/day) |  |  |  |  |  |  |  |
| None | 299 | 28 | 11,500 | 30 |  | 11,799 | 30 |
| <20 | 651 | 60 | 22,553 | 59 |  | 23,204 | 59 |
| ≥20 | 90 | 8 | 3,027 | 8 |  | 3,117 | 8 |
| Unknown | 45 | 4 | 1,390 | 4 |  | 1,435 | 4 |
|  |  |  |  |  |  |  |  |
| Physical activity (strenuous and moderate) (hours/week) |  |  |  |  |  |  |  |
| <3.8 | 392 | 36 | 12,496 | 32 |  | 12,888 | 33 |
| 3.8-5.9 | 335 | 31 | 12,653 | 33 |  | 12,988 | 33 |
| >6.0 | 346 | 32 | 12,790 | 33 |  | 13,136 | 33 |
| Unknown | 12 | 1 | 531 | 1 |  | 543 | 1 |
|  |  |  |  |  |  |  |  |
| Age at menopause (years) |  |  |  |  |  |  |  |
| 10-39 | 66 | 6 | 2,843 | 7 |  | 2,909 | 7 |
| 40-49 | 265 | 24 | 10,048 | 26 |  | 10,313 | 26 |
| 50-54 | 354 | 33 | 12,243 | 32 |  | 12,597 | 32 |
| 55-59 | 157 | 14 | 5,161 | 13 |  | 5,318 | 13 |
| 60-70 | 19 | 2 | 654 | 2 |  | 673 | 2 |
| Unknown | 224 | 21 | 7,521 | 20 |  | 7,745 | 20 |
|  |  |  |  |  |  |  |  |
| Hormone therapy use |  |  |  |  |  |  |  |
| Never | 188 | 17 | 7,576 | 20 |  | 7,764 | 20 |
| Ever | 800 | 74 | 26,571 | 69 |  | 27,371 | 69 |
| Unknown | 97 | 9 | 4,323 | 11 |  | 4,420 | 11 |
|  |  |  |  |  |  |  |  |
| Age at menarche (years) |  |  |  |  |  |  |  |
| <12 | 258 | 24 | 8,827 | 23 |  | 9,085 | 23 |
| 12-13 | 629 | 58 | 21,829 | 57 |  | 22,458 | 57 |
| ≥14 | 187 | 17 | 7,352 | 19 |  | 7,539 | 19 |
| Unknown/Never | 11 | 1 | 463 | 1 |  | 473 | 1 |
|  |  |  |  |  |  |  |  |
| Age at first full-term pregnancy (years) |  |  |  |  |  |  |  |
| No full-term pregnancy | 248 | 23 | 8,617 | 22 |  | 8,865 | 22 |
| <25 years | 280 | 26 | 10,380 | 27 |  | 10,660 | 27 |
| 25-29 | 325 | 30 | 11,843 | 31 |  | 12,168 | 31 |
| ≥30 | 221 | 20 | 7,064 | 18 |  | 7,285 | 18 |
| Unknown | 11 | 1 | 566 | 1 |  | 577 | 1 |
|  |  |  |  |  |  |  |  |
| Breast feeding history (months) |  |  |  |  |  |  |  |
| Never pregnant | 196 | 18 | 6,480 | 17 |  | 6,676 | 17 |
| Pregnancy, but no live birth | 52 | 5 | 2,095 | 5 |  | 2,147 | 5 |
| Never breastfed | 173 | 16 | 5,593 | 15 |  | 5,766 | 15 |
| 1-5 months | 168 | 15 | 6,460 | 17 |  | 6,628 | 17 |
| 6-11 | 176 | 16 | 5,612 | 15 |  | 5,788 | 15 |
| ≥12 | 306 | 28 | 11,525 | 30 |  | 11,831 | 30 |
| Unknown | 14 | 1 | 705 | 2 |  | 719 | 2 |
|  |  |  |  |  |  |  |  |
| Diabetes |  |  |  |  |  |  |  |
| No | 973 | 90 | 34,863 | 91 |  | 35,836 | 91 |
| Yes | 101 | 9 | 3,241 | 8 |  | 3,342 | 8 |
| Unknown | 11 | 1 | 366 | 1 |  | 377 | 1 |
|  |  |  |  |  |  |  |  |
| Chronic obstructive pulmonary disease (COPD) |  |  |  |  |  |  |  |
| No | 1012 | 93 | 35,753 | 93 |  | 36,765 | 93 |
| Yes | 28 | 3 | 1,094 | 3 |  | 1,122 | 3 |
| Unknown | 45 | 4 | 1,623 | 4 |  | 1,668 | 4 |
|  |  |  |  |  |  |  |  |
| Parkinson’s disease |  |  |  |  |  |  |  |
| No | 1038 | 96 | 36,723 | 95 |  | 37,761 | 95 |
| Yes | 7 | 1 | 203 | 1 |  | 210 | 1 |
| Unknown | 40 | 4 | 1,544 | 4 |  | 1,584 | 4 |
|  |  |  |  |  |  |  |  |
| Depression |  |  |  |  |  |  |  |
| No | 831 | 77 | 29,617 | 77 |  | 30,448 | 77 |
| Yes | 211 | 19 | 7,149 | 19 |  | 7,360 | 19 |
| Unknown | 43 | 4 | 1,704 | 4 |  | 1,747 | 4 |
|  |  |  |  |  |  |  |  |
| Chronic fatigue syndrome |  |  |  |  |  |  |  |
| No | 1009 | 93 | 36,019 | 94 |  | 37,028 | 94 |
| Yes | 29 | 3 | 810 | 2 |  | 839 | 2 |
| Unknown | 47 | 4 | 1,641 | 4 |  | 1,688 | 4 |
|  |  |  |  |  |  |  |  |
| Lupus |  |  |  |  |  |  |  |
| No | 1023 | 94 | 36,242 | 94 |  | 37,265 | 94 |
| Yes | 8 | 1 | 308 | 1 |  | 316 | 1 |
| Unknown | 54 | 5 | 1,920 | 5 |  | 1,974 | 5 |
|  |  |  |  |  |  |  |  |
| Inflammatory bowel disease/Crohn’s disease |  |  |  |  |  |  |  |
| No | 993 | 92 | 35,360 | 92 |  | 36,353 | 92 |
| Yes | 51 | 5 | 1,492 | 4 |  | 1,543 | 4 |
| Unknown | 41 | 4 | 1,618 | 4 |  | 1,659 | 4 |
|  |  |  |  |  |  |  |  |
| Multiple sclerosis |  |  |  |  |  |  |  |
| No | 1039 | 96 | 36,748 | 96 |  | 37,787 | 96 |
| Yes | 8 | 1 | 201 | 1 |  | 209 | 1 |
| Unknown | 38 | 4 | 1,521 | 4 |  | 1,559 | 4 |
|  |  |  |  |  |  |  |  |
| Pain medication use |  |  |  |  |  |  |  |
| No | 987 | 91 | 35,114 | 91 |  | 36,101 | 91 |
| Yes | 65 | 6 | 2,108 | 5 |  | 2,173 | 5 |
| Unknown | 33 | 3 | 1,248 | 3 |  | 1,281 | 3 |
|  |  |  |  |  |  |  |  |
| Non-Steroidal Anti-Inflammatory Drug (NSAID) use |  |  |  |  |  |  |  |
| No | 404 | 37 | 15,049 | 39 |  | 15,453 | 39 |
| Yes | 623 | 57 | 21,021 | 55 |  | 21,644 | 55 |
| Unknown | 58 | 5 | 2,400 | 6 |  | 2,458 | 6 |
|  |  |  |  |  |  |  |  |
| Marital status |  |  |  |  |  |  |  |
| Married | 660 | 61 | 23,806 | 62 |  | 24,466 | 62 |
| Divorced/Separated | 164 | 15 | 5,517 | 14 |  | 5,681 | 14 |
| Widowed | 152 | 14 | 5,625 | 15 |  | 5,777 | 15 |
| Never married | 78 | 7 | 2,355 | 6 |  | 2,433 | 6 |
| Unknown | 31 | 3 | 1,167 | 3 |  | 1,198 | 3 |
|  |  |  |  |  |  |  |  |
| Annual Household income ($) |  |  |  |  |  |  |  |
| < $50,000 | 151 | 14 | 4,430 | 12 |  | 4,581 | 12 |
| 50,000-74,999 | 205 | 19 | 7,601 | 20 |  | 7,806 | 20 |
| 75,000-99,999 | 185 | 17 | 6,859 | 18 |  | 7,044 | 18 |
| 100,000-149,999 | 192 | 18 | 7,010 | 18 |  | 7,202 | 18 |
| ≥150,000 | 114 | 11 | 4,502 | 12 |  | 4,616 | 12 |
| Unknown | 238 | 22 | 8,068 | 21 |  | 8,306 | 21 |
|  |  |  |  |  |  |  |  |
| Education level |  |  |  |  |  |  |  |
| At least Master’s Degree | 539 | 50 | 19,209 | 50 |  | 19,748 | 50 |
| Bachelors Degree | 347 | 32 | 12,431 | 32 |  | 12,778 | 32 |
| Less than Bachelors | 5 | <1 | 214 | 1 |  | 219 | 1 |
| Unknown | 194 | 18 | 6,616 | 17 |  | 6,810 | 17 |
